# Redetermination of tricalcium trilanthanum penta­kis(ortho­borate) from single-crystal data

**DOI:** 10.1107/S1600536808014785

**Published:** 2008-05-21

**Authors:** Tianyong Zhou, Ning Ye

**Affiliations:** a Fujian Institute of Research on the Structure of Matter, Chinese Academy of Sciences, Fuzhou, Fujian 350002, People’s Republic of China

## Abstract

Single crystals of the title compound, Ca_3_La_3_(BO_3_)_5_, were obtained by spontaneous nucleation from a high-temperature melt. The crystal structure of Ca_3_La_3_(BO_3_)_5_ has been determined previously from X-ray powder data [Zhang, Liang, Chen, He & Xu (2001). *J. Alloys Compd*, **327**, 96–99]. The present refinement shows a significant improvement in terms of the precision of the geometric parameters and the correct determination of the absolute configuration in space group *P*6_3_ *mc *with all atoms refined with anisotropic displacement parameters. The structure consists of isolated BO_3_ triangles and distorted [CaO_8_] and [LaO_10_] polyhedra. Except for one O atom, all other atoms are situated on special positions: La, all O and one B atom on mirror planes, and two B atoms with site symmetry 3*m*.

## Related literature

For phase equilibria in the system La_2_O_3_—CaO—B_2_O_3_, see: Zhang *et al.* (2001*a* ▶). For a previous structure analysis of Ca_3_La_3_(BO_3_)_5_ based on X-ray powder diffraction data, see: Zhang *et al.* (2001*b* ▶). For non-linear optical (NLO) applications of borate crystals containing triangular BO_3_ anions, see: Chen *et al.* (1999 ▶). For a review of the geometry of the BO_3_ group, see: Zobetz (1982 ▶). For the potential applications of Ca_3_La_3_(BO_3_)_5_ for photoluminescence, see: Zhang *et al.* (2005 ▶); Han *et al.* (2007 ▶).

## Experimental

### 

#### Crystal data

Ca_3_La_3_(BO_3_)_5_
*M* *_r_* = 831.02
Hexagonal, 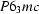

*a* = 10.530 (3) Å
*c* = 6.398 (2) Å
*V* = 614.4 (3) Å^3^
*Z* = 2
Mo *K*α radiation
μ = 11.59 mm^−1^
*T* = 293 (2) K
0.22 × 0.12 × 0.10 mm

#### Data collection

Rigaku Mercury CCD diffractometer
Absorption correction: multi-scan (*CrystalClear*; Rigaku, 2000 ▶) *T*_min_ = 0.206, *T*_max_ = 0.304
4065 measured reflections
534 independent reflections
534 reflections with *I* > 2σ(*I*)
*R*_int_ = 0.035

#### Refinement

*R*[*F* ^2^ > 2σ(*F* ^2^)] = 0.012
*wR*(*F* ^2^) = 0.030
*S* = 0.89
534 reflections
53 parameters
1 restraint
Δρ_max_ = 0.41 e Å^−3^
Δρ_min_ = −0.59 e Å^−3^
Absolute structure: Flack (1983 ▶), 236 Friedel pairs
Flack parameter: −0.03 (3)

### 

Data collection: *CrystalClear* (Rigaku, 2000 ▶); cell refinement: *CrystalClear*; data reduction: *CrystalClear*; program(s) used to solve structure: *SHELXS97* (Sheldrick, 2008 ▶); program(s) used to refine structure: *SHELXL97* (Sheldrick, 2008 ▶); molecular graphics: *DIAMOND* (Brandenburg, 2004 ▶); software used to prepare material for publication: *enCIFer* (Allen *et al.*, 2004 ▶).

## Supplementary Material

Crystal structure: contains datablocks global, I. DOI: 10.1107/S1600536808014785/wm2179sup1.cif

Structure factors: contains datablocks I. DOI: 10.1107/S1600536808014785/wm2179Isup2.hkl

Additional supplementary materials:  crystallographic information; 3D view; checkCIF report

## Figures and Tables

**Table:** 

Ca1—O4^i^	2.3139 (13)
Ca1—O1^ii^	2.376 (3)
Ca1—O3	2.382 (4)
Ca1—O1^iii^	2.662 (3)
La1—O1^iv^	2.501 (2)
La1—O4^v^	2.516 (4)
La1—O2^vi^	2.6639 (15)
La1—O3^vii^	2.8112 (8)
B1—O4	1.358 (6)
B1—O1^i^	1.384 (3)
B2—O2^viii^	1.374 (3)
B3—O3	1.389 (3)

**Table:** 

O4—B1—O1^i^	119.7 (2)
O1^i^—B1—O1^x^	120.6 (4)
O2^viii^—B2—O2^xi^	120
O3^ix^—B3—O3	120

Symmetry codes: (i) 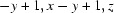; (ii) 
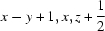
; (iii) 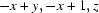; (iv) 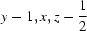; (v) 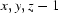; (vi) 
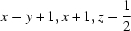
; (vii) 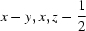; (viii) 
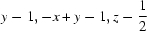
; (ix) 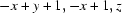; (x) 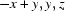; (xi) 
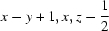
.

**Fig. 1.**
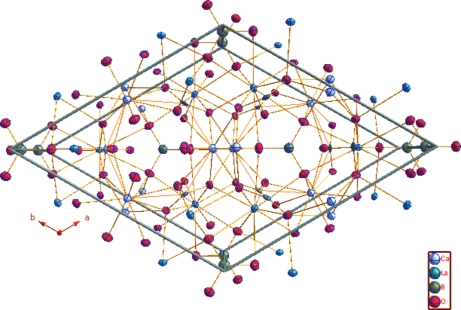
The structure of (I) in a projection approximatly along the [001] direction with displacement ellipsoids drawn at the 85% probability level.

**Fig. 2.**
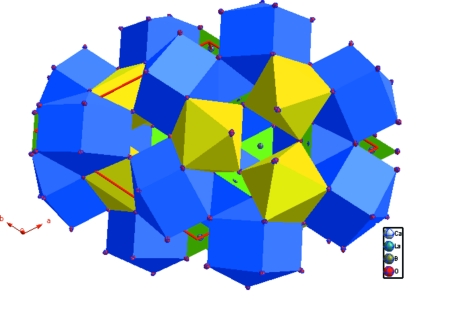
Packing diagram of the structure of (I). [CaO8] polyhedra are yellow, [LaO10] polyhedra are blue and [BO3] units are green.

## supplementary crystallographic information

### Comment

Borate crystals containing parallel aligned BO_3_ anions are predicted to have large nonlinear optical (NLO) coefficients, moderate birefringence and wide transparency in the UV-region. Therefore they are considered to be good candidates for NLO applications (Chen, 1999). The title compound Ca_3_La_3_(BO_3_)_5_, (I), has been investigated previously by Zhang *et al.* (2001a) during analysis of phase equilibria in the system La_2_O_3_—CaO—B_2_O_3_, and NLO and luminescent properties of this material have also been reported (Zhang, 2005; Han, 2007). The crystal structure of Ca_3_La_3_ (BO_3_)_5_ was originally determined from X-ray powder diffraction data in conjunction with IR spectroscopy (Zhang *et al.*, 2001b).

The structure of compound (I) can be described in terms of BO_3_ triangles and complex irregular [CaO_8_] and [LaO_10_] polyhedra. Each of the three crystallographically different B atoms is coordinated to three O atoms to form planar BO_3_ triangles. The B—O bond lengths range from 1.384 (3) to 1.389 (3) Å, which is in good agreement with the results of geometric studies of the BO_3_ unit (Zobetz, 1982). Two of the three BO_3_ groups exhibit 3*m* symmetry, and the third BO_3_ group has *m* symmetry with O–B–O angles very close to 120°. The La^3+^ cations are 10-fold coordinated by oxygen atoms with  La—O bond lengths ranging from 2.501 (2) to 2.812 (2) Å. The [LaO_10_] polyhedra are connected to each other and to the borate groups by sharing corners and edges forming a three-dimensional network with channels running parallel to [001]. In these channels the Ca^2+^ cations are situated and are surrounded by eight oxygen atoms with Ca—O bond lengths ranging from 2.3139 (13) to 2.662 (3) Å (Table 1).

### Experimental

Single crystals of compound (I) were grown using a LiBO_2_-containing flux. The composition of the mixture for crystal growth was 1:1:4:3 of CaCO_3_ (Sinopharm Regent, AR), La_2_O_3_ (Materials, 99.8%), H_3_BO_3_ (Sinopharm Regent, 99.99%), and Li_2_CO_3_ (Sinopharm Reagent, AR). The mixture was heated in a platinum crucible to 1373 K, held at this temperature for several hours, and then cooled at a rate of 10 K/h from 1373 to 873 K. The remaining solified flux attached to the crystals was readily dissolved in water. Crystals with an average size of 0.5 mm and mostly rod shaped habit were obtained.

### Refinement

The present study confirms the basic structural features determined from the previous investigation by Zhang *et al.* (2001*b*) with a much higher precesion and with all displacement parameters refined anisotropically.

### Crystal data

**Table:**

Ca_3_La_3_(BO_3_)_5_	*Z* = 2
*M**_r_* = 831.02	*F*_000_ = 752
Hexagonal, *P*6_3_*m**c*	*D*_x_ = 4.492 Mg m^−^^3^
Hall symbol: P 6c -2c	Mo *K*α radiation λ = 0.71073 Å
*a* = 10.530 (3) Å	Cell parameters from 1909 reflections
*b* = 10.530 (3) Å	θ = 2.2–27.5º
*c* = 6.398 (2) Å	µ = 11.59 mm^−^^1^
α = 90º	*T* = 293 (2) K
β = 90º	Rod, colourless
γ = 120º	0.22 × 0.12 × 0.10 mm
*V* = 614.4 (3) Å^3^	

### Data collection

**Table:**

Rigaku Mercury CCD diffractometer	534 independent reflections
Radiation source: Sealed Tube	534 reflections with *I* > 2σ(*I*)
Monochromator: Graphite Monochromator	*R*_int_ = 0.035
Detector resolution: 14.6306 pixels mm^-1^	θ_max_ = 27.5º
*T* = 293(1) K	θ_min_ = 2.2º
CCD_Profile_fitting scans	*h* = −13→13
Absorption correction: multi-scan(CrystalClear; Rigaku, 2000)	*k* = −13→13
*T*_min_ = 0.206, *T*_max_ = 0.304	*l* = −8→7
4065 measured reflections	

### Refinement

**Table:**

Refinement on *F*^2^	Secondary atom site location: difference Fourier map
Least-squares matrix: full	*w* = 1/[σ^2^(*F*_o_^2^) + (0.02*P*)^2^ + 1.5843*P*] where *P* = (*F*_o_^2^ + 2*F*_c_^2^)/3
*R*[*F*^2^ > 2σ(*F*^2^)] = 0.012	(Δ/σ)_max_ < 0.001
*wR*(*F*^2^) = 0.030	Δρ_max_ = 0.41 e Å^−^^3^
*S* = 0.89	Δρ_min_ = −0.59 e Å^−^^3^
534 reflections	Extinction correction: SHELXL97 (Sheldrick, 2008), Fc^*^=kFc[1+0.001xFc^2^λ^3^/sin(2θ)]^-1/4^
53 parameters	Extinction coefficient: 0.0632 (12)
1 restraint	Absolute structure: Flack (1983), 236 Friedel pairs
Primary atom site location: structure-invariant direct methods	Flack parameter: −0.03 (3)

### Special details

**Table:**

Geometry. All e.s.d.'s (except the e.s.d. in the dihedral angle between two l.s. planes) are estimated using the full covariance matrix. The cell e.s.d.'s are taken into account individually in the estimation of e.s.d.'s in distances, angles and torsion angles; correlations between e.s.d.'s in cell parameters are only used when they are defined by crystal symmetry. An approximate (isotropic) treatment of cell e.s.d.'s is used for estimating e.s.d.'s involving l.s. planes.
Refinement. Refinement of *F*^2^ against ALL reflections. The weighted *R*-factor *wR* and goodness of fit *S* are based on *F*^2^, conventional *R*-factors *R* are based on *F*, with *F* set to zero for negative *F*^2^. The threshold expression of *F*^2^ > σ(*F*^2^) is used only for calculating *R*-factors(gt) *etc*. and is not relevant to the choice of reflections for refinement. *R*-factors based on *F*^2^ are statistically about twice as large as those based on *F*, and *R*- factors based on ALL data will be even larger.

### Fractional atomic coordinates and isotropic or equivalent isotropic displacement parameters (Å^2^)

**Table:**

	*x*	*y*	*z*	*U*_iso_*/*U*_eq_	
Ca1	0.47334 (5)	0.52666 (5)	0.76261 (15)	0.00673 (19)	
La1	0.156065 (12)	0.843935 (12)	0.08229 (8)	0.00493 (11)	
B1	0.1989 (3)	0.8011 (3)	0.5473 (8)	0.0049 (10)	
B2	0	0	0.2435 (15)	0.0086 (17)	
B3	0.6667	0.3333	0.598 (3)	0.0092 (19)	
O1	0.6272 (3)	0.9278 (2)	0.4462 (4)	0.0067 (5)	
O2	0.07534 (16)	0.92466 (16)	0.7399 (6)	0.0097 (7)	
O3	0.59052 (16)	0.40948 (16)	0.5984 (8)	0.0083 (6)	
O4	0.22657 (17)	0.77343 (17)	0.7443 (5)	0.0066 (6)	

### Atomic displacement parameters (Å^2^)

**Table:**

	*U*^11^	*U*^22^	*U*^33^	*U*^12^	*U*^13^	*U*^23^
Ca1	0.0060 (3)	0.0060 (3)	0.0073 (4)	0.0023 (3)	−0.0001 (2)	0.0001 (2)
La1	0.00442 (12)	0.00442 (12)	0.00474 (14)	0.00129 (9)	0.00003 (8)	−0.00003 (8)
B1	0.0052 (15)	0.0052 (15)	0.007 (3)	0.0044 (18)	−0.0006 (10)	0.0006 (10)
B2	0.011 (3)	0.011 (3)	0.003 (4)	0.0057 (13)	0	0
B3	0.009 (2)	0.009 (2)	0.009 (6)	0.0046 (11)	0	0
O1	0.0069 (10)	0.0056 (10)	0.0073 (11)	0.0029 (9)	−0.0014 (10)	0.0016 (9)
O2	0.0090 (12)	0.0090 (12)	0.0124 (16)	0.0055 (14)	−0.0005 (7)	0.0005 (7)
O3	0.0101 (10)	0.0101 (10)	0.0067 (17)	0.0065 (11)	0.0005 (9)	−0.0005 (9)
O4	0.0067 (11)	0.0067 (11)	0.0055 (16)	0.0026 (13)	−0.0014 (7)	0.0014 (7)

### Geometric parameters (Å, °)

**Table:**

Ca1—O4^i^	2.3139 (13)	La1—O1^i^	2.678 (3)
Ca1—O4^ii^	2.3139 (13)	La1—O3^xiv^	2.8112 (8)
Ca1—O1^iii^	2.376 (3)	La1—O3^xv^	2.8112 (8)
Ca1—O1^iv^	2.376 (3)	La1—B2^xvi^	3.028 (3)
Ca1—O3	2.382 (4)	La1—B1	3.076 (5)
Ca1—O3^v^	2.444 (5)	B1—O4	1.358 (6)
Ca1—O1^ii^	2.662 (3)	B1—O1^i^	1.384 (3)
Ca1—O1^vi^	2.662 (3)	B1—O1^xiii^	1.384 (3)
Ca1—B1^i^	2.858 (4)	B1—Ca1^i^	2.858 (4)
Ca1—B1^ii^	2.858 (4)	B1—Ca1^ii^	2.858 (4)
Ca1—Ca1^v^	3.3435 (11)	B2—O2^xvii^	1.374 (3)
Ca1—Ca1^vii^	3.3435 (11)	B2—O2^xviii^	1.374 (3)
La1—O1^viii^	2.501 (2)	B2—O2^xix^	1.374 (3)
La1—O1^ix^	2.501 (2)	B2—La1^xx^	3.028 (3)
La1—O4^x^	2.516 (4)	B2—La1^i^	3.028 (3)
La1—O2^x^	2.639 (3)	B2—La1^xxi^	3.028 (3)
La1—O2^xi^	2.6639 (15)	B3—O3^xxii^	1.389 (3)
La1—O2^xii^	2.6639 (15)	B3—O3^xxiii^	1.389 (3)
La1—O1^xiii^	2.678 (3)	B3—O3	1.389 (3)
			
O4^i^—Ca1—O4^ii^	93.58 (15)	O1^ix^—La1—O3^xiv^	116.83 (10)
O4^i^—Ca1—O1^iii^	151.80 (11)	O4^x^—La1—O3^xiv^	64.52 (12)
O4^ii^—Ca1—O1^iii^	80.01 (9)	O2^x^—La1—O3^xiv^	122.29 (12)
O4^i^—Ca1—O1^iv^	80.01 (9)	O2^xi^—La1—O3^xiv^	155.42 (13)
O4^ii^—Ca1—O1^iv^	151.80 (11)	O2^xii^—La1—O3^xiv^	121.81 (10)
O1^iii^—Ca1—O1^iv^	92.72 (12)	O1^xiii^—La1—O3^xiv^	88.50 (10)
O4^i^—Ca1—O3	126.18 (10)	O1^i^—La1—O3^xiv^	65.77 (12)
O4^ii^—Ca1—O3	126.18 (10)	O1^viii^—La1—O3^xv^	116.83 (9)
O1^iii^—Ca1—O3	77.64 (10)	O1^ix^—La1—O3^xv^	69.51 (8)
O1^iv^—Ca1—O3	77.64 (10)	O4^x^—La1—O3^xv^	64.52 (12)
O4^i^—Ca1—O3^v^	73.69 (10)	O2^x^—La1—O3^xv^	122.29 (12)
O4^ii^—Ca1—O3^v^	73.69 (10)	O2^xi^—La1—O3^xv^	121.81 (10)
O1^iii^—Ca1—O3^v^	78.17 (9)	O2^xii^—La1—O3^xv^	155.42 (13)
O1^iv^—Ca1—O3^v^	78.17 (9)	O1^xiii^—La1—O3^xv^	65.77 (12)
O3—Ca1—O3^v^	144.65 (19)	O1^i^—La1—O3^xv^	88.50 (10)
O4^i^—Ca1—O1^ii^	56.39 (9)	O3^xiv^—La1—O3^xv^	50.66 (12)
O4^ii^—Ca1—O1^ii^	112.85 (10)	O4—B1—O1^i^	119.7 (2)
O1^iii^—Ca1—O1^ii^	151.00 (8)	O4—B1—O1^xiii^	119.7 (2)
O1^iv^—Ca1—O1^ii^	86.53 (8)	O1^i^—B1—O1^xiii^	120.6 (4)
O3—Ca1—O1^ii^	73.88 (10)	O2^xvii^—B2—O2^xviii^	120.00 (0)
O3^v^—Ca1—O1^ii^	129.62 (7)	O2^xvii^—B2—O2^xix^	120.00 (0)
O4^i^—Ca1—O1^vi^	112.85 (10)	O2^xviii^—B2—O2^xix^	120.00 (0)
O4^ii^—Ca1—O1^vi^	56.39 (9)	O3^xxii^—B3—O3^xxiii^	120.00 (0)
O1^iii^—Ca1—O1^vi^	86.53 (8)	O3^xxii^—B3—O3	120.00 (0)
O1^iv^—Ca1—O1^vi^	151.00 (8)	O3^xxiii^—B3—O3	120.00 (0)
O3—Ca1—O1^vi^	73.88 (10)	B1^ii^—O1—Ca1^xv^	147.6 (3)
O3^v^—Ca1—O1^vi^	129.62 (7)	B1^ii^—O1—La1^xxiv^	114.0 (3)
O1^ii^—Ca1—O1^vi^	80.50 (11)	Ca1^xv^—O1—La1^xxiv^	94.81 (8)
O1^viii^—La1—O1^ix^	138.96 (12)	B1^ii^—O1—Ca1^i^	83.5 (2)
O1^viii^—La1—O4^x^	73.88 (6)	Ca1^xv^—O1—Ca1^i^	82.95 (8)
O1^ix^—La1—O4^x^	73.88 (6)	La1^xxiv^—O1—Ca1^i^	87.75 (8)
O1^viii^—La1—O2^x^	71.80 (6)	B1^ii^—O1—La1^ii^	92.9 (2)
O1^ix^—La1—O2^x^	71.80 (6)	Ca1^xv^—O1—La1^ii^	89.98 (9)
O4^x^—La1—O2^x^	64.64 (11)	La1^xxiv^—O1—La1^ii^	111.47 (9)
O1^viii^—La1—O2^xi^	121.07 (8)	Ca1^i^—O1—La1^ii^	160.08 (10)
O1^ix^—La1—O2^xi^	71.30 (9)	B2^xxv^—O2—La1^xxvi^	123.0 (5)
O4^x^—La1—O2^xi^	137.71 (9)	B2^xxv^—O2—La1^xxvii^	91.42 (19)
O2^x^—La1—O2^xi^	82.07 (7)	La1^xxvi^—O2—La1^xxvii^	107.69 (7)
O1^viii^—La1—O2^xii^	71.30 (9)	B2^xxv^—O2—La1^xxviii^	91.42 (19)
O1^ix^—La1—O2^xii^	121.07 (8)	La1^xxvi^—O2—La1^xxviii^	107.69 (7)
O4^x^—La1—O2^xii^	137.71 (9)	La1^xxvii^—O2—La1^xxviii^	135.45 (14)
O2^x^—La1—O2^xii^	82.07 (7)	B3—O3—Ca1	154.0 (8)
O2^xi^—La1—O2^xii^	53.07 (13)	B3—O3—Ca1^vii^	118.3 (8)
O1^viii^—La1—O1^xiii^	137.03 (9)	Ca1—O3—Ca1^vii^	87.71 (10)
O1^ix^—La1—O1^xiii^	83.72 (6)	B3—O3—La1^xxix^	94.64 (7)
O4^x^—La1—O1^xiii^	129.92 (8)	Ca1—O3—La1^xxix^	86.76 (7)
O2^x^—La1—O1^xiii^	146.79 (6)	Ca1^vii^—O3—La1^xxix^	85.94 (9)
O2^xi^—La1—O1^xiii^	68.76 (8)	B3—O3—La1^iii^	94.64 (7)
O2^xii^—La1—O1^xiii^	92.23 (9)	Ca1—O3—La1^iii^	86.76 (7)
O1^viii^—La1—O1^i^	83.72 (6)	Ca1^vii^—O3—La1^iii^	85.94 (9)
O1^ix^—La1—O1^i^	137.03 (9)	La1^xxix^—O3—La1^iii^	169.80 (13)
O4^x^—La1—O1^i^	129.92 (8)	B1—O4—Ca1^ii^	98.91 (12)
O2^x^—La1—O1^i^	146.79 (6)	B1—O4—Ca1^i^	98.91 (12)
O2^xi^—La1—O1^i^	92.23 (9)	Ca1^ii^—O4—Ca1^i^	145.78 (15)
O2^xii^—La1—O1^i^	68.76 (8)	B1—O4—La1^xxvi^	127.4 (3)
O1^xiii^—La1—O1^i^	53.37 (10)	Ca1^ii^—O4—La1^xxvi^	96.00 (9)
O1^viii^—La1—O3^xiv^	69.51 (8)	Ca1^i^—O4—La1^xxvi^	96.00 (9)

Symmetry codes: (i) −*y*+1, *x*−*y*+1, *z*; (ii) −*x*+*y*, −*x*+1, *z*; (iii) *x*−*y*+1, *x*, *z*+1/2; (iv) −*x*+1, −*x*+*y*, *z*+1/2; (v) −*x*+1, −*y*+1, *z*+1/2; (vi) *x*, *x*−*y*+1, *z*; (vii) −*x*+1, −*y*+1, *z*−1/2; (viii) *y*−1, *x*, *z*−1/2; (ix) −*x*+1, −*y*+2, *z*−1/2; (x) *x*, *y*, *z*−1; (xi) *x*−*y*+1, *x*+1, *z*−1/2; (xii) *y*−1, −*x*+*y*, *z*−1/2; (xiii) −*x*+*y*, *y*, *z*; (xiv) *x*−*y*, *x*, *z*−1/2; (xv) *y*, −*x*+*y*+1, *z*−1/2; (xvi) *x*, *y*+1, *z*; (xvii) *y*−1, −*x*+*y*−1, *z*−1/2; (xviii) *x*−*y*+1, *x*, *z*−1/2; (xix) −*x*, −*y*+1, *z*−1/2; (xx) −*x*+*y*−1, −*x*, *z*; (xxi) *x*, *y*−1, *z*; (xxii) −*x*+*y*+1, −*x*+1, *z*; (xxiii) −*y*+1, *x*−*y*, *z*; (xxiv) −*x*+1, −*y*+2, *z*+1/2; (xxv) −*x*, −*y*+1, *z*+1/2; (xxvi) *x*, *y*, *z*+1; (xxvii) *y*−1, −*x*+*y*, *z*+1/2; (xxviii) *x*−*y*+1, *x*+1, *z*+1/2; (xxix) *y*, −*x*+*y*, *z*+1/2.
